# Immobilisation of Cellobiose Dehydrogenase and Laccase on Chitosan Particles as a Multi-Enzymatic System for the Synthesis of Lactobionic Acid

**DOI:** 10.3390/jfb14070383

**Published:** 2023-07-21

**Authors:** Justyna Sulej, Wiktoria Piątek-Gołda, Marcin Grąz, Katarzyna Szałapata, Piotr Waśko, Ewa Janik-Zabrotowicz, Monika Osińska-Jaroszuk

**Affiliations:** 1 Department of Biochemistry and Biotechnology, Institute of Biological Sciences, Maria Curie-Sklodowska University, 19 Akademicka St., 20-033 Lublin, Poland; wiktoria.piatek-97@wp.pl (W.P.-G.); marcin.graz@mail.umcs.pl (M.G.); katarzyna.szalapata@mail.umcs.pl (K.S.); 2 Department of Plant Physiology and Biophysics, Institute of Biological Sciences, Maria Curie-Sklodowska University, 19 Akademicka St., 20-033 Lublin, Poland; piotr.wasko@mail.umcs.pl; 3 Core Facility of Biospectroscopy, Institute of Biological Sciences, Maria Curie-Sklodowska University, 19 Akademicka St., 20-033 Lublin, Poland; ewa.janik-zabrotowicz@mail.umcs.pl; 4 Department of Cell Biology, Institute of Biological Sciences, Maria Curie-Sklodowska University, 19 Akademicka St., 20-033 Lublin, Poland

**Keywords:** chitosan, microspheres, immobilisation, lactobionic acid, cellobiose dehydrogenase, laccase, genipin

## Abstract

Lactobionic acid (LBA) is a bioactive compound that has become increasingly popular in medicine in recent years due to its unique properties. This chemical can be formed via the enzymatic oxidation of lactose using fungal oxidoreductive enzymes. This study aimed to intensify the synthesis of LBA using immobilised enzymes (cellobiose dehydrogenase from *Phanerochaete chrysosporium* (PchCDH) and laccase from *Cerrena unicolor* (CuLAC)) on chitosan microspheres. We used three different crosslinking agents: genipin, glutaraldehyde, and polyethyleneimine to activate the chitosan. The FTIR and CellDrop techniques were used to characterise the activated microspheres. Quantitative (HPLC) and qualitative (TLC) methods were used to determine the obtained LBA. The results show that the type of activator used influences the efficiency of the binding of the enzyme to the matrix. Furthermore, the amount of LBA formed depends on the type of system used. The use of a system in which one of the enzymes is immobilised on a PEI-activated carrier (PchCDH) and the other is free (CuLAC) proved to be the most optimal, as it yielded almost 100% conversion of lactose to lactobionic acid. Summarising the data obtained the following: lactobionic acid immobilised on chitosan microspheres has great potential for medical applications.

## 1. Introduction

Lactobionic acid (LBA, 4-O-β-D-galactopyranosyl-D-gluconic acid), i.e., a derivative of lactose, has diverse functional properties which are especially interesting for the medical, cosmetic, pharmaceutical, food, and chemical industries [1,2]. The primary commercial application of LBA is its use as a component of cold-storage solutions applied to stabilise organs before transplantation [3]. In the biomedical context, metal-chelating properties are very important for the reduction of oxidative damage to tissues caused by some metal ions, e.g., iron, during the storage and preservation of organs [4]. Additionally, lactobionic acid has been shown to inhibit hypothermia-induced cellular swelling in various tissues; additionally, it does not penetrate cell membranes at low temperatures due to its size and three-dimensional structure [5]. LBA is also widely used in the dermatology and cosmetic industries in preparations designed to treat such diseases as atopic dermatitis, rosacea, and seborrheic dermatitis because it is a weak skin irritant, which is important for preventing skin problems. This new substance with powerful antioxidant, wound healing, moisturising, anti-aging, and keratinising effects is increasingly being used in cosmetics [6]. Lactobionic acid can also be used as a prebiotic substance for stimulation of the growth of probiotic bacteria contained in synbiotic preparations applied as functional food supplements [7]. There are various methods for producing LBA (chemical, electrochemical, catalytic, and enzymatic approaches or fermentation); however, green biocatalytic approaches using enzymes are of great interest due to their high selectivity, specificity, and mild reaction conditions. Enzymatic catalysis requires purified lactose-oxidising enzymes, mainly derived from fungi, to convert lactose to LBA [1]. Enzymatic oxidation is a very effective method of LBA synthesis, especially in the form based on cellobiose dehydrogenase, laccase, and appropriate mediators creating an indirect flavoenzyme regeneration system (biotransformation system).

Cellobiose dehydrogenase (CDH; EC1.1.99.18) is an extracellular flavoenzyme produced by a great number of rot fungi and contains two cofactors (flavin and heme) connected via a flexible proteolysis-sensitive linker. CDH catalyses the oxidation reaction of di- and oligosaccharides linked by β-1-4-glucosidic bonds, such as cellobiose and lactose, to corresponding lactones spontaneously converted to aldonic acids [8]. The enzyme can transfer electrons to various redox mediators such as 2,6-dichloro-indophenol (DCIP), 1,4-benzoquinone, 2,2-azino-bis (3-ethylbenzothiazoline-6-sulphonic acid) (ABTS), and metal ions [9].

Laccase (LAC, EC 1.10.3.2), p-diphenol oxidase, needs to be added simultaneously into the system as a regenerative enzyme to oxidise redox mediators that serve as an electron acceptor for CDH [10,11]. This enzyme is included in multicopper oxidases (MCOs) and can oxidise organic and inorganic compounds such as mono-, di, poly, amino-, and methoxyphenols and several aromatic amines [12] with concurrent reduction of molecular oxygen to water. Laccases are widespread in the world of living organisms such as plants, insects, fungi, and bacteria.

The increasing interest in immobilised enzymes as a tool for a variety of applications has prompted our team to further study LBA synthesis in multi-enzymatic systems. The design of immobilised enzyme systems using various support materials is often applied in biotechnological and biomedical processes [13,14,15]. Immobilisation of enzymes helps to simplify the process setup, compared to the use of native biocatalysts [16,17]. A very important issue in the immobilisation process is the selection of the carrier supports and the method of binding the enzyme. The use of appropriate support matrices protects the three-dimensional structure of the enzyme from unfavourable reaction conditions and therefore enables the immobilised enzyme to exhibit enhanced biocatalytic activity [15].

Increasing numbers of research findings are being published today on the immobilization of enzymes on microparticles with high specific surface areas and many active sites available for the fixation of the enzyme molecules. Additionally, because the support particles are smaller, there is less internal diffusion resistance [18].

Enzymes can be immobilized via a variety of techniques, which can be broadly categorized as physical (weak interactions between the support and the enzyme) and chemical (covalent bonds between the support and the enzyme form) [19].

In our study, we used chitosan microparticles as a support for the immobilisation of both oxidoreductases used in the preparation of the LBA synthesis system.

Chitosan is a natural hydrophilic polymer with high primary amino groups having excellent properties, such as nontoxicity, biocompatibility, chemical reactivity, and allowing easy enzyme fixation [20,21]. This polymer is a perfect foundation for enzyme immobilization since it is soluble in a mildly acidic aqueous solution, mechanically strong, resistant to chemical deterioration, widely commercially available, and inexpensive [22].

Chitosan has been widely used for the immobilisation of various enzymes. For this purpose, it can be activated through the derivatisation of the amine group with glutaraldehyde. The exposed aldehyde moiety of glutaraldehyde can then react with an amino group of enzymes for immobilisation thereof [15]. However, due to its toxicity, we also tested genipin and PEI in our research, which are often used as cross-linking agents in biomedical applications. Genipin is a reagent isolated from *Gardenia jasminoides* fruit extracts. It has good crosslinking properties [23,24], while its acute toxicity is low [25,26,27].

The reaction between genipin and primary and secondary amines is particularly beneficial for polymer chemistry and many other biological applications [28].

The manufacturing of chitosan materials, biological scaffolds for tissue engineering, and chitosan and protein nanoparticles for controlled drug delivery are the principal applications [29].

The main aim of this work was to construct various bienzymatic systems based on cellobiose dehydrogenase from *Phanerochaete chrysosporium*, laccase from *Cerrena unicolor*, and ABTS as a mediator for the synthesis of lactobionic acid. The enzymes were immobilised on chitosan microspheres activated with glutaraldehyde, genipin, and PEI. The chitosan microspheres were characterised in terms of the morphological structure, and FTIR spectra showed changes in Amide I, which is regarded to be characteristic of the *α*-helical structure in the protein.

In the studies shown in this work, we chose to use microparticles due to the possibilities they offer. The use of microspheres allows for the application of the immobilized enzyme in various industries, including cosmetology and medicine [30,31]. Chitosan microspheres are used as drug delivery systems [32] and transporters of bioactive substances such as vitamin E [33].

The novelty of our research is the immobilization of fungal oxidoreductive enzymes, which can have applications in biotechnology, e.g., in biomedicine. We have shown that an immobilized enzyme system can successfully be used to produce lactobionic acid (LBA). In our previous studies, we have shown that LBA has antioxidant and antimicrobial properties, which indicates that it can be successfully used in biotechnology [34].

## 2. Materials and Method

### 2.1. Microorganisms

The fungal strains, *Phanerochaete chrysosporium* (FCL236) and *Cerrena unicolor* (FCL139), were obtained from culture collections maintained at cooperating universities. Namely, FCL236 was obtained from the Tokyo Agricultural University, while FCL139 was obtained from the University of Regensburg in Germany. To secure their proper storage and availability, both strains were then deposited in the fungal collection located at the Department of Biochemistry and Biotechnology of the Maria Curie-Sklodowska University in Lublin, Poland. The fungi were genetically identified and their corresponding nucleotide sequences were registered in the GenBank database. The accession numbers assigned to their nucleotide sequences are DQ056858 for FCL139 and FJ594058 for FCL236.

### 2.2. Materials

The chitosan powder (medium molecular weight, 75 to 85% deacetylated), the poly(ethyleneimine) 50% *v/v* solution (PEI), and the glutaraldehyde (25% (*v*/*v*)) aqueous solution were obtained from Sigma Aldrich (Steinheim, Germany). Genipin (GEN, >98.00%, wt%) was supplied by Pol-Aura (Morąg, Poland). All of the substances required for the investigation were of the highest purity and analytical grade (98%) Sigma Aldrich (Steinheim, Germany), Merck (Darmstadt, Germany), VWR (Vienna, Austria), Bio-Rad (Warsaw, Poland), or BioMaxima (Lublin, Poland) were the suppliers of the media components and other chemicals. Deionized water was used for the production of all aqueous solutions.

### 2.3. Enzyme Preparation

The producers of fungal oxidoreductases (cellobiose dehydrogenase; CDH and laccase; LAC) used in the study were white rot fungi *P. chrysosporium* (FCL236) as a source of CDH and *C. unicolor* (FCL139) as a source of LAC. Enzymes were obtained and isolated according to the methods described in previously published works [34,35,36].

### 2.4. Analysis of Proteins and Enzyme Activity

Cellobiose dehydrogenase activity was determined using the Baminger method with modifications. The absorbance decrease was measured for 60 s at λ = 520 nm at 30 °C (ɛ520 = 6.8 mM^−1^ cm^−1^) in the presence of 2,6-dichloroindophenol (DCIP) and lactose [34,37].

Laccase activity was determined with the method developed by Grzywnowicz and Leonowicz with modifications.

Syringaldazine (4-hydroxy-3,5-dimethoxybenzaldehyde azine) at a concentration of 0.5 mM was used as an acceptor to assess laccase activity. At = 525 nm and 25 °C, the rise in absorbance was recorded for 60 s [30,34].

Protein concentrations (CDH and LAC) were determined with the Bradford method with bovine serum albumin (BSA) as a standard [38].

### 2.5. Immobilisation of Cellobiose Dehydrogenase on Chitosan Microspheres

#### 2.5.1. Preparation of Chitosan Microspheres

The chitosan solution was prepared by dissolving 3 g of chitosan powder in 100 mL of a 2% acetic acid solution (*v*/*v*) under magnetic stirring for 5 h at a temperature of 50 °C until complete dissolution and then degassing by ultrasonication. The prepared solution was added with a pipette to a glass beaker containing McIlveine buffer pH = 8.0. The system was mixed on a magnetic stirrer (800 rpm) for 30 min. The solution was brought to a pH of 12.0 with 1 M NaOH. Once the pH was determined, the solution was agitated for 15 min on a magnetic stirrer with a speed of 400 rpm. The microspheres were then stored overnight at 4 °C. The following day, the chitosan microspheres were washed using deionised water until neutrality. The carrier was stored underwater in the fridge until activation.

#### 2.5.2. Activation

The chitosan microspheres were prepared in three variants. Each was activated with a different crosslinking agent: 5% glutaraldehyde (GA), 0.5% genipin (GEN), and 0.5% polyethyleneimine (PEI). The crosslinking agent concentrations were chosen based on preliminary studies. The microspheres were treated with the appropriate crosslinking agent solution and then incubated for one hour at room temperature (30 min with 100 rpm shaking and 30 min without shaking). After activation, the microspheres were washed several times with deionised water.

#### 2.5.3. Immobilisation

Chitosan microspheres (2 g) were mixed with a solution containing CDH (0.3 mg/mL) and LAC (0.02 mg/mL) (2 mL). After 3 h of agitation at 25 °C, 350 rpm, the mixture was refrigerated overnight. The chitosan spheres with bound enzymes were then separated and washed three times with deionized water to remove unbound enzymes.

##### Activity of Immobilised Enzymes

The activity of immobilised CuLAC was measured in Mc Ilveine buffer, pH 5.5, at 30 °C using syringaldazine as a substrate. The activity of immobilised PchCDH was measured in 0.1 M acetate buffer, pH 4.5, at 30 °C using lactose as a substrate and DCIP as a co-substrate.

Activity (AKT_imm_) was calculated from Equation (1) and expressed in U per gram wet weight of the carrier (U/g), which is the amount of enzyme capable of oxidising 1 μmol of a substrate, where ΔAmin is the absorbance per minute, Vt is the total sample volume (3 mL), ε is the molar absorption coefficient of a substrate (for CuCDH = 6.5 × 10^−4^ M^−1^ cm^−1^) or co-substrate (for PchCDH = 6.8 × 10^−4^ M^−1^ cm^−1^), t is the reaction time (60 s), and m is the mass of the carrier used in the reaction (g).

(1) $${AKT}_{imm}=\frac{{AKT}_{min}\;*\;V_{t}}{\varepsilon \;*\;t\;*\;m}$$

The calculations were based on the work of Wlizło et al. [39].

##### Efficiency of Enzyme Immobilisation

The quantity of immobilised enzymes (*E*_imm_) was calculated from the difference between the concentration of enzyme protein on the carrier (*E*_applied_) and the concentration of protein present in the eluates obtained after elution of the unbound enzyme from the carrier (*E*_unbound_) and expressed in mg of protein per g wet weight of carrier according to Equation (2). The protein immobilisation efficiency and activity yield was presented as a percentage (%) based on Equation (3) [39,40].

(2) $$E_{imm}\;\left(\frac{mg}{g}\right)=E_{applied}-E_{unbound}$$

(3) $$Yield\;\left(\%\right)=\frac{E_{imm}}{E_{applied}}\times 100\%$$

### 2.6. Characterisation of Chitosan Microspheres

#### 2.6.1. CellDrop

The evaluation of the microsphere particle size distribution and the determination of their basic morphological structure were carried out using the CellDrop counter (Wilmington, NC, USA) with Software Version v2.1.4. Immediately before the measurement, 5% aqueous solutions of microspheres were mixed for several seconds on a laboratory vortex to obtain a homogeneous suspension, and then 40 µL of the sample was taken and dispensed manually into the measuring aperture of the apparatus. The measurement was carried out in the bright-field mode with the measuring chamber set at a height of 400 µm. The following algorithm was used for particle counting: minimum detectable particle diameter 6 µm, maximum detectable particle diameter 30 µm, small cell mode on. The measurement was performed in 10 independent repetitions for each of the tested variants.

#### 2.6.2. Fourier-Transform Infrared Spectroscopy (FTIR)

Measurements of infrared spectroscopy were carried out with the use of a Vertex 70 FTIR spectrometer (Bruker, Billerica, MA, USA). All measurements were performed using a ZnSe crystal plate (10 internal reflections, 45° cut) in the spectral range between 4000–600 cm^−1^ in the attenuated total reflection configuration (ATR). All spectra were recorded as Fourier-transformed and 16 averaged scans with a spectral resolution of 2 cm^−1^ per data point. Dry N_2_ gas was used for purification of the measurement chamber 30 min before and during recordings. Each of the samples was deposited as a colloid on the ATR crystal and dried with a weak flux of N_2_ for not less than 30 min. Spectral analyses were carried out with the Grams/AI software (ThermoGalactic Industries, Waltham, MA, USA). All spectra were normalised by their area for easier comparison. The differential spectra of the Amide I band were obtained by subtracting the spectra of immobilised chitosan microspheres with PEI, GEN, or GA from the spectra of the same samples with the added CDH or LAC enzymes. Changes in the Amide I band (1700–1590 cm^−1^) were calculated by deconvolution of the differential spectra. Deconvolution of this band was obtained with mathematical decomposition and the use of Gauss curves. The R^2^ factor of the fitted curve was not lower than 0.99.

### 2.7. Enzymatic Oxidation of Lactose and Synthesis of Lactobionic Acid (LBA)

The synthesis of lactobionic acid (LBA) was based on our previous studies with minor modifications. The multi-enzymatic system consisted of the PchCDH and CuLAC enzymes in a 1:1 ratio, 50 mM lactose (substrate), and ABTS as a mediator of the redox reaction. We tested a combination of free and immobilised enzymes as well as two enzymes immobilised independently on chitosan microspheres activated with different crosslinking agents. The reaction was carried out for 24 h at 50 °C. After incubation, the samples were ultrafiltrated through centrifuge concentrators (Vivaspin 500) using a polyethylene sulfone (PES) membrane with a 10 kDa cut-off (Sartorius, Göttingen, Germany).

The reaction mixture was analysed for the amount of lactobionic acid formed in the samples. For this purpose, qualitative (TLC) and quantitative (HPLC) analyses were used [34,41].

#### 2.7.1. Use of Thin-Layer Chromatography to Determine LBA

The approach suggested by Kiryu was used for thin-layer liquid chromatography (TLC), with modifications presented in detail in an earlier study [34].

Lactobionic acid was determined qualitatively using Kaisel Gel 60 TLC plates from Merck in Darmstadt, Germany, together with the ethyl acetate, acetic acid, and water developing method.

The plate was separated and sprayed with H_2_SO_4_ and methanol. Then, it was heated to 150 °C to disclose spots that indicated the presence of lactose and lactobionic acid [42].

#### 2.7.2. Determination of LBA Using High-Performance Liquid Chromatography

Lactobionic acid (LBA) and lactose concentrations were determined using high-performance liquid chromatography (HPLC) with an Agilent Infinity 1260 system featuring refractive index (RID) and diode array (DAD) detectors. The HPLC system had a Bio-Rad Aminex HPX-87H column maintained at a temperature of 50 °C. The mobile phase consisted of 0.45 mM H_2_SO_4_ and was delivered at a flow rate of 0.7 mL/min. The injection time was set to 20 s. The procedure was performed according to previous studies [34].

### 2.8. Statistical Analysis

The results presented in this study represent the mean ± SD obtained from three separate experiments (n = 3). Mean and standard deviation calculations were performed using one-way ANOVA analysis with Statgraphics Online software. Tukey’s multiple range test was then used to compare the means. Microsoft Office 365 Excel was used to calculate the data. Statistical significance was determined by considering *p*-values less than or equal to 0.05.

## 3. Results and Discussion

### 3.1. Immobilisation of Enzymes on Chitosan Microspheres

**Figure 1:**
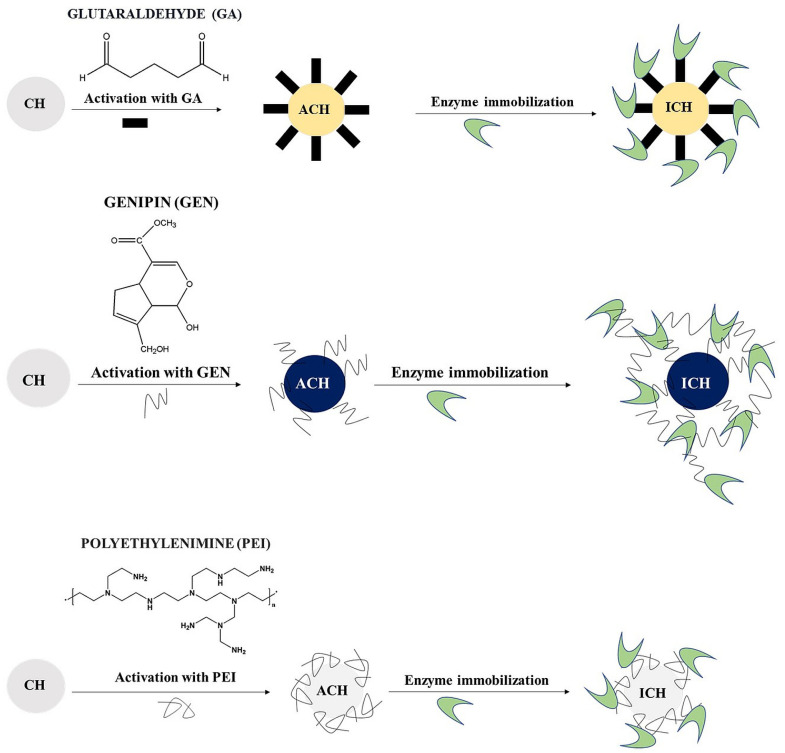
Steps of enzyme immobilisation on chitosan microspheres. CH—chitosan microspheres, ACH—activated chitosan microspheres, ICH—immobilised microspheres.

In this study, we tested the effect of the crosslinking agent on the efficiency of enzyme binding to chitosan microspheres. Additionally, we aimed to determine the enzymatic activity of immobilised biocatalysts. We used three different crosslinking agents (5% glutaraldehyde (GA), 0.5% genipin (GEN), and 0.5% polyethyleneimine (PEI), which enabled the enzyme to bind to the carrier (Figure 1). The tested enzymes (cellobiose dehydrogenase CDH or laccase LAC) were immobilised on chitosan microspheres activated with one of the three tested crosslinking agents.

Table 1 shows the immobilisation yields for the different variants, including the immobilisation efficiency determined using the indirect and direct methods. When laccase was used, the best results were achieved using PEI, where the enzymatic activity of the biocatalyst was completely preserved (100% efficiency). In the case of GA and GEN, the enzyme was deactivated, and no enzymatic activity directly on the carrier was observed. Considering the results obtained in the experiment for PchCDH, it is worth mentioning that all the crosslinking agents tested proved to be effective. The immobilisation efficiency in this variant ranged from 44.89% to 100% for the different crosslinking agents. The best results were obtained using 5% GEN (100% efficacy).

Enzyme immobilisation is widely used in industry instead of the application of free biocatalysts [15,43,44]. The use of different crosslinking agents helps to develop systems that will have the highest efficiency. In our study, we used three crosslinking agents to activate chitosan microspheres: genipin, glutaraldehyde, and polyethyleneimine. The GA compound is commonly used as a crosslinking agent [35,45,46,47]. However, its use in industry is limited due to its irritant and toxic properties towards humans and aquatic organisms [48,49]. The application of compounds with high biocompatibility and no negative impact on humans or the environment has been proven crucial by the scientific world. One such substance is genipin. Our team was the first to decide to use GEN as a cross-linking agent in the immobilisation of CDH. We opted to use genipin due to its low toxicity and good crosslinking properties [50,51]. Our results show that it is possible to use genipin as an activating agent for chitosan microspheres resulting in binding approximately 98% of the protein (CDH) and retaining 100% of the enzymatic activity on the carrier. Despite the 35% LAC immobilisation efficiency, we did not observe the activity of this enzyme directly on the carrier.

It is worth mentioning that other research teams have also used genipin as a matrix-activating compound for immobilisation, and their results confirm our assumptions about its potential use in the immobilisation of cellobiose dehydrogenase. Moreover, there are several literature reports on biomedical applications of genipin [52,53,54].

The third crosslinking agent used in our study is a cationic macromolecule commonly used in gene transfer protocols and therapies with high transfection efficiency both in vitro and in vivo [55,56,57,58].

Polyethyleneimine is used as a crosslinking agent by many research teams around the world [59,60,61,62]. An example of the use of PEI to activate a substrate for immobilisation is the system using urease to detect urea [63] or peroxidase to identify paracetamol [64]. The present results show that PEI can be successfully used for the activation of chitosan microspheres with the maintenance of the enzymatic activity of the immobilised biocatalyst. Our team’s research has focused on the use of polyethyleneimine as a crosslinking agent to facilitate enzyme attachment, which can later find applications in various industries and medicine.

### 3.2. Characterisation of Chitosan Microspheres

#### 3.2.1. CellDrop

In order to characterise the morphology and particle size of the chitosan microsphere preparations, an innovative method based on measurements in the bright-field mode on the CellDrop apparatus was used. Based on the image obtained in the bright-field mode, the CellDrop apparatus counts the number of particles present in the field of view using the introduced measurement algorithm. The proposed method allows combining the advantages of simultaneous generation of an image and determining the average particle size for each of the tested variants based on measurements of representative samples. The most popular standard methods used for imaging and characterisation of microspheres and nanoparticles are light microscopy, laser diffraction analysis using a Mastersizer device, X-ray diffraction (XRD), Raman spectroscopy, transmission electron microscopy (TEM), scanning electron microscopy (SEM), and atomic force microscopy (AFM) [32,65,66,67,68]. Nevertheless, non-invasive imaged-based cell/molecule counting methods have recently been gaining in popularity due to their speed and low cost of measurements [69,70,71,72].

**Figure 2:**
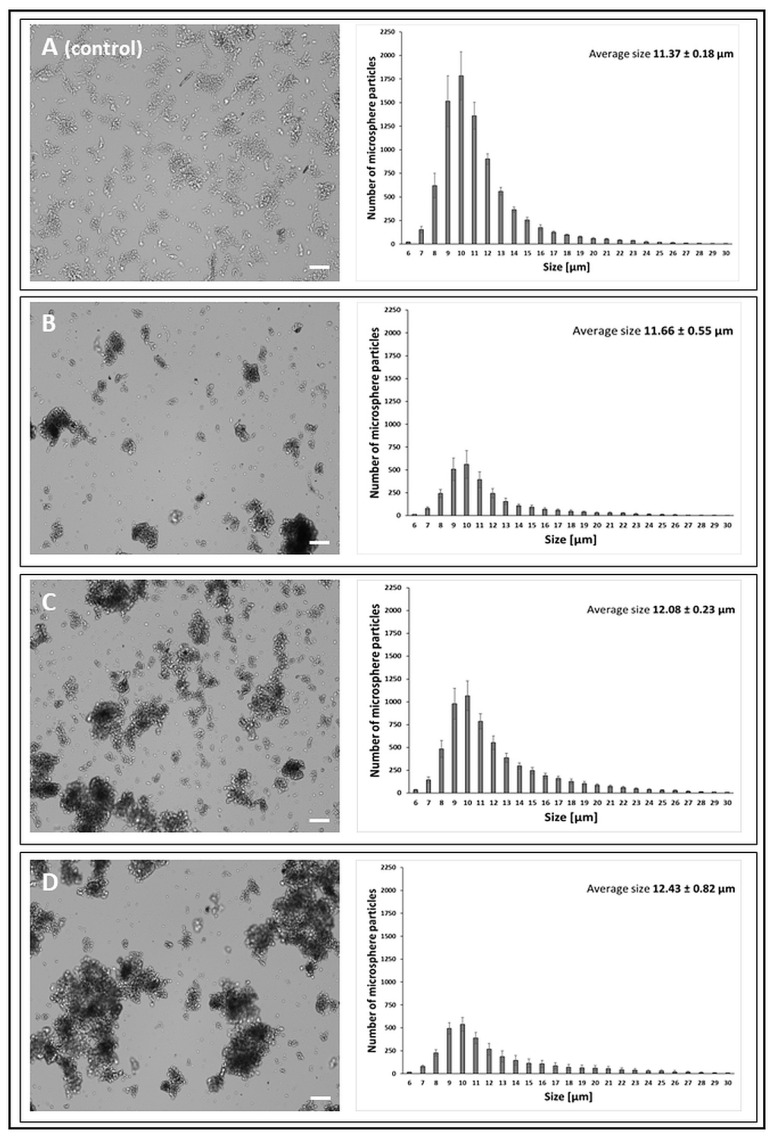
Morphology of chitosan microspheres (bright-field mode, CellDrop cell counter) and size distribution of microsphere particles obtained with the GA (glutaraldehyde) cross-linker. The scale bar is 100 µm. (**A**)—chitosan particles (*control*); (**B**)—chitosan activated with 5% GA; (**C**)—chitosan activated with 5% GA and with immobilised CDH; (**D**)—chitosan activated with 5% GA and with immobilised LAC.

Based on the obtained results of the average particle size, it can be concluded that the chitosan activation process carried out with the use of three cross-linking compounds induced changes in the diameter of the microspheres. The greatest differences were observed in the case of GA activation, where the average particle size of the microsphere gradually increased with the successive stages of surface modification (Figure 2)—chitosan activated with GA 11.66 µm, chitosan activated with GA and immobilised CDH 12.08 µm, and chitosan activated with GA and immobilised LAC 12.43 µm. At the same time, the microspheres obtained with the use of GA showed high structural stiffness. Taking into account the possibility of spatial cross-linking between the enzyme molecules and immobilisation mainly through lysine residues [73], the microsphere preparations obtained with the use of GA may have reduced the enzymatic activity.

**Figure 3:**
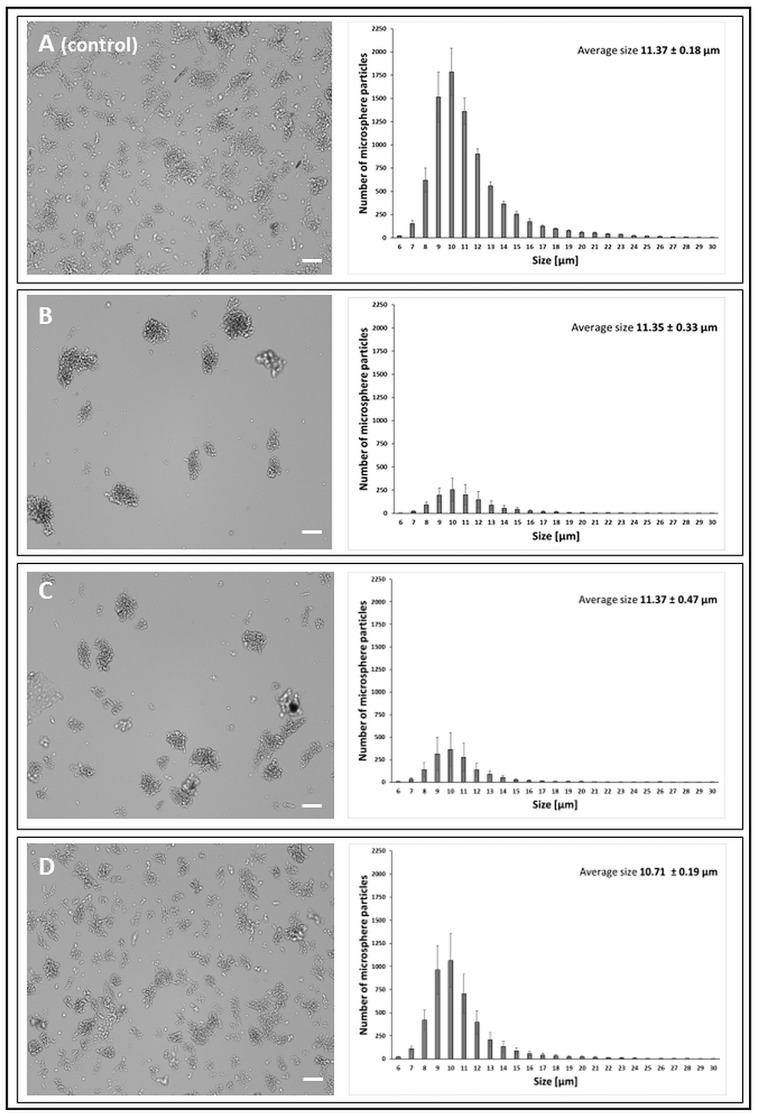
Morphology of chitosan microspheres (bright-field mode, CellDrop cell counter) and size distribution of microsphere particles obtained with the PEI (polyethyleneimine) cross-linker. The scale bar is 100 µm. (**A**)—chitosan particles (*control*); (**B**)—chitosan activated with 0.5% PEI; (**C**)—chitosan activated with 0.5% PEI and with immobilised CDH; (**D**)—chitosan activated with 0.5% PEI and with immobilised LAC.

**Figure 4:**
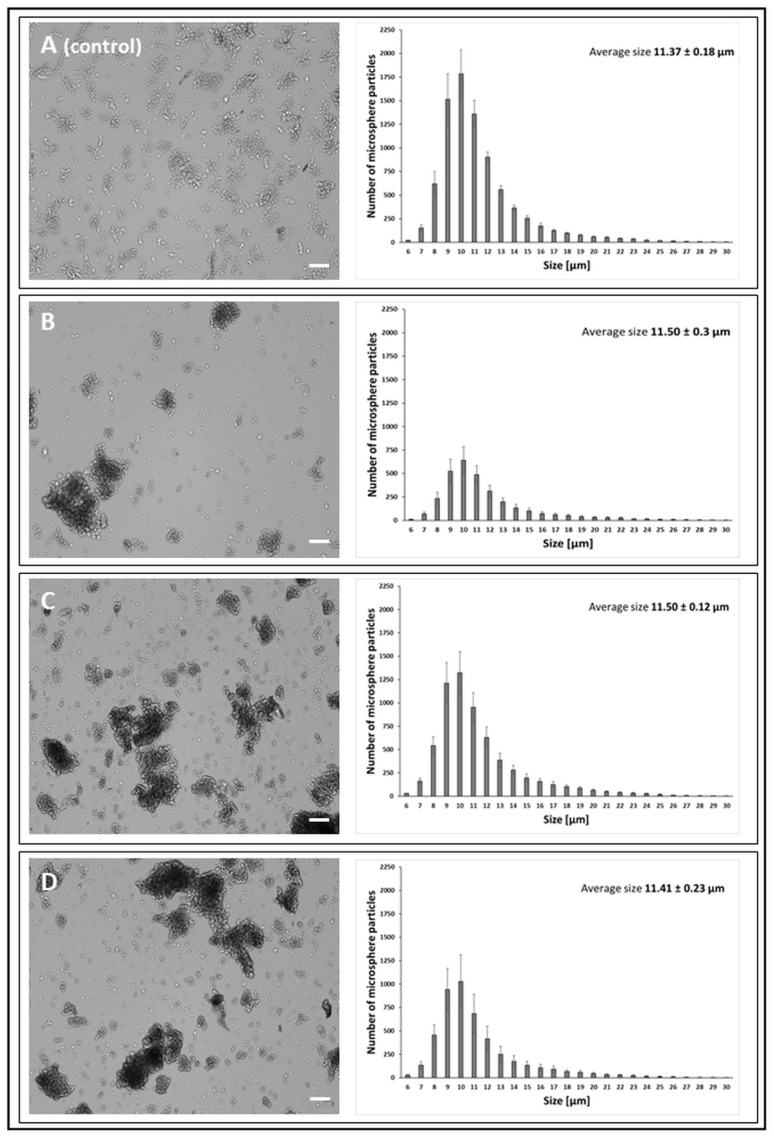
Morphology of chitosan microspheres (bright-field mode, CellDrop cell counter) and size distribution of microsphere particles obtained with the GEN (genipin) cross-linker. The scale bar is 100 µm. (**A**)—chitosan particles (*control*); (**B**)—chitosan activated with 0.5% GEN; (**C**)—chitosan activated with 0.5% GEN and with immobilised CDH; (**D**)—chitosan activated with 0.5% GEN and with immobilised LAC.

Microspheres activated with PEI and with immobilised LAC and CDH were characterised by the highest dispersion and formed the smallest aggregates (Figure 3). In addition, the aggregates formed by these microspheres in the aqueous environment showed the greatest structural flexibility, in contrast to the microsphere preparations activated with the use of GA and GEN. Most aggregates composed of more than six particles were formed by GEN-activated microsphere preparations (Figure 4) numerical data not shown on charts. The type of the cluster and the number of particle aggregates in the cluster are shown in the Supplementary Material in Figures S2–S4.

#### 3.2.2. FTIR Analysis

**Figure 5:**
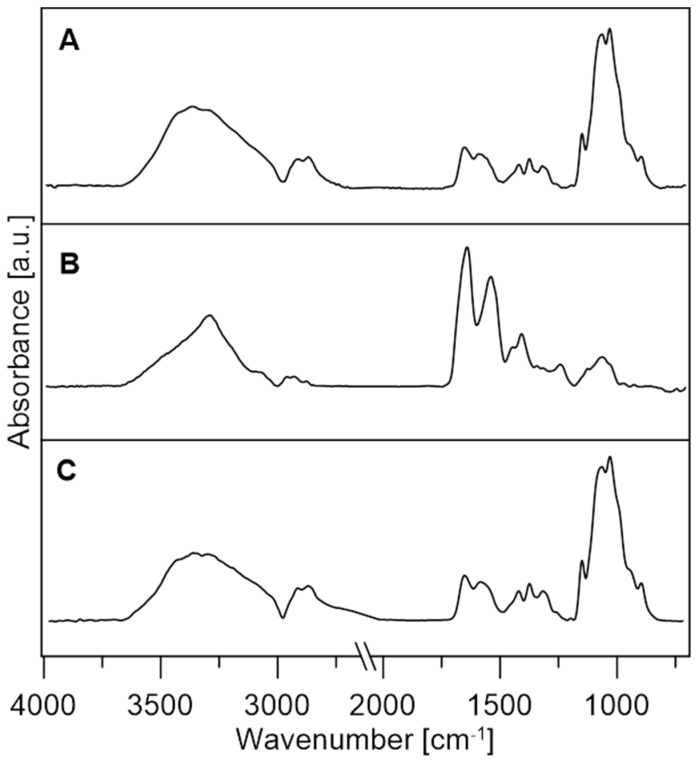
IR absorbance spectra of laccase (**A**), cellobiose dehydrogenase (**B**), and chitosan microspheres (**C**).

The influence of the immobilising substance and the background on the enzyme structure provides important information not only about its binding to the surface of the background material but also about enzyme activity. The FTIR spectra of the tested enzymes and chitosan microspheres deposited on the ZnSe crystal are presented in Figure 5. It can be seen that the spectra of laccase and chitosan are very similar because their chemical structure and functional groups are similar. The spectra consist of characteristic polypeptide bands [74,75,76].

**Figure 6:**
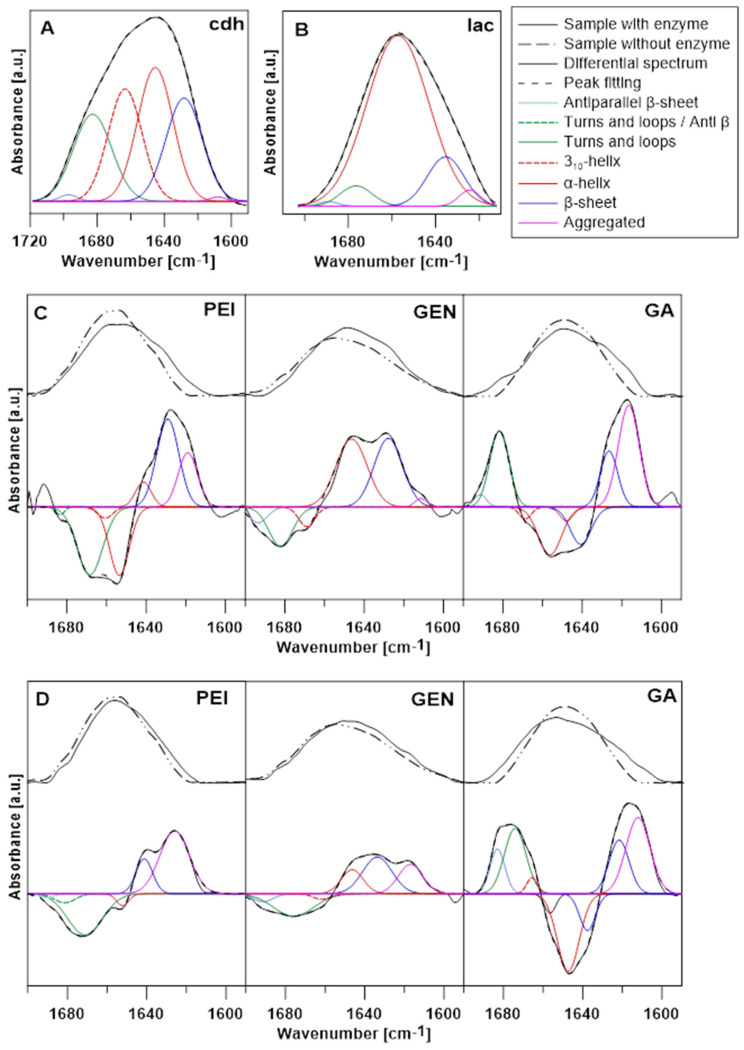
Structure analysis of the Amide I band of CDH (**A**) and laccase (**B**); the continuous black line marks the Amide I band of the enzymes, the dashed line marks estimated peak fitting. The colour lines mark various protein secondary structures. Influence of the immobilising factor (PEI, GEN, and GA) on Amide I structure changes in CDH (**C**) and laccase (**D**). The upper panels show the Amide I band of samples containing and non-containing enzymes; the lower panels show differential spectra and their Gaussian deconvolution.

Amide I (1720–1580 cm^−1^) is the most characteristic and widely analysed band present in protein and enzyme spectra. The structure of this band is composed of various components reflecting the secondary structure of protein molecules. The analyses of the Amide I band of the CDH and laccase enzymes are presented in Figure 6, panels A and B. The α-helix accounting for 29.78% of the Amide I band area is the largest component in the CDH secondary structure, and turns and loops, β-sheet, and the 3_10_-helix structure have significant contributions to this band, representing an area above 20%. The secondary structure of laccase is composed mostly of an α-helix with 77.96% of the band area (Table 2) [77,78]. Table 3 shows the secondary structure changes caused by activating substances.

### 3.3. Lactose Oxidation by Enzymes and Lactobionic Acid (LBA) Production

Fifteen enzyme systems were tested using both free and immobilised enzymes as well as various crosslinking agents. A sample containing free enzymes was the control.

The tested systems were labelled according to the following pattern:

✓ PchCDH/GEN/GA/PEI-chitosan activated with genipin, glutaraldehyde, or polyethyleneimine with immobilised CDH.
✓ CuLAC/GEN/GA/PEI-chitosan activated with genipin, glutaraldehyde, or polyethyleneimine with immobilised LAC.

We used the following combinations in the study:

1. PchCDH/GA + CuLAC,
2. PchCDH/PEI + CuLAC,
3. PchCDH/GEN + CuLAC,
4. CuLAC/GA + PchCDH,
5. CuLAC/PEI + PchCDH,
6. CuLAC/GEN + PchCDH,
7. PchCDH/GEN + CuLAC/GEN,
8. PchCDH/GEN + CuLAC/PEI,
9. PchCDH/GEN + CuLAC/GA,
10. PchCDH/GA + CuLAC/GEN,
11. PchCDH/GA + CuLAC/PEI,
12. PchCDH/GA + CuLAC/GA,
13. PchCDH/PEI + CuLAC/GEN,
14. PchCDH/PEI + CuLAC/PEI,
15. PchCDH/PEI + CuLAC/GA.

In this assay, the effect of the immobilisation process on the efficiency of LBA synthesis was tested. We based the experiment on our previous study with some modifications because we aimed to create a system comprising immobilised enzymes. The enzymatic systems were tested at 50 °C, and incubation was carried out for 24 h. ABTS was used as a redox mediator. The presence of lactobionic acid in the tested sample was confirmed by TLC and HPLC chromatography. To show the effect of the immobilisation process on the efficiency of LBA synthesis, we checked the amount of acid formed by the enzymatic reaction with free enzymes and determined the efficiency of conversion of lactose to lactobionic acid.

#### 3.3.1. Thin-Layer Liquid Chromatography (TLC) Analysis

The qualitative assessment of the obtained lactobionic acid was conducted employing the thin-layer chromatography (TLC) method, as depicted in Figure S1. This analytical approach offered supplementary confirmation of the presence of synthesized LBA in the examined samples.

We followed the method described in our previous work. We used commercial LBA at a concentration of 20 mM and 50 mM lactose as standards [34]. Visualisation of the resulting spots allowed us to carry out qualitative determination of the presence or absence of lactobionic acid in the reaction mixture. In addition, by comparing previously published results, the correctness of the chromatographic separation was confirmed [34,42].

#### 3.3.2. High-Performance Liquid Chromatography Analysis

For this purpose, we used high-performance liquid chromatography (HPLC) to determine the concentration of lactobionic acid produced. We analysed all the tested systems using HPLC, and the results are shown in the table below (Table 4). The best results were obtained in systems where cellobiose dehydrogenase was immobilised and laccase was present in the reaction mixture. 

The highest concentration (43.01 mM), with approximately 93% efficiency of conversion of lactose to LBA during the enzymatic oxidation process, was observed in the system with CDH immobilised on polyethyleneimine-activated support and with free LAC. A conversion rate of 45% was achieved in a control system that contained both enzymes in their unbound form.

Systems in which both enzymes were immobilised were not as efficient. The biocatalysts immobilised on PEI microspheres facilitated the synthesis of LBA at a level of 9.81 mM, corresponding to approximately 22% efficiency of conversion of lactose to lactobionic acid.

The yield of lactobionic acid synthesis in the current study is about two times higher than in our previous study. In this experiment, we were able to synthesise as much as 43 mM LBA, giving 93% efficiency of conversion of lactose to LBA, whereas previously we obtained a maximum of 21 mM LBA, which accounted for 43% of the conversion of lactose to lactobionic acid [34].

PchCDH/PEI + CuLAC combination was the optimal system for the synthesis of lactobionic acid. Immobilisation of PchCDH, which catalyses the oxidation of lactose to LBA, proved to be of key importance for the development of the system with the highest efficiency of lactose conversion to LBA. Free LAC facilitated the complete use of this enzyme as a biocatalyst for ABTS regeneration. 

Compared to our previous studies, a clear upward trend in synthesised LBA can be observed in this study, confirming that the immobilisation process increases the efficiency of the lactobionic acid conversion process.

The use of immobilised enzymes for lactobionic acid synthesis is a novelty in science. Yang’s team was the first to decide to publish results on the effect of enzyme immobilisation on the efficiency of LBA synthesis. They used CDH from *Aspergillus fumigatus* and LAC from *Trametes* sp. The enzymes were separately immobilised on glutaraldehyde-modified magnetic chitosan (MCS) beads and the conversion efficiency was 100% [41].

## 4. Conclusions

The present study tested and compared for the first time the influence of three different crosslinking agents on the performance of immobilisation of fungal oxidoreductases. Determination of the shape and size of the microspheres after the activation process made it possible to determine the influence of the crosslinking agents on the matrix used for the immobilisation process. Fourier-transform infrared (FTIR) spectroscopy provided information about the change in the protein structure after binding to the microspheres. The development of systems for the production of lactobionic acid showed the possibility of using the studied enzymes in various industries. The quantitative and qualitative characterisation of the resulting products was facilitated successively by thin-layer liquid chromatography (TLC) and high-performance liquid chromatography (HPLC). The present study demonstrated that lactobionic acid can be obtained via synthesis using immobilised enzymes, with the amount of LBA depending on the system used. The use of a system in which one of the enzymes is immobilised on a PEI-activated carrier (PchCDH) and the other is free (CuLAC) proved to be the most optimal, thus giving 93.34% conversion of lactose to lactobionic acid.

## Figures and Tables

**Table 1:** Efficiency of cellobiose dehydrogenase (CDH) and laccase (LAC) immobilisation on the chitosan microspheres activated by the different crosslinking agents (glutaraldehyde GA, genipin GEN, and polyethyleneimine PEI).

Sample	Applied Protein [mg/g Carrier]	Immobilised Protein [mg/g Carrier]	Protein Yield [%]	Activity of Enzyme Bound with Chitosan [U/g Carrier]	Activity Yield [%]
CDH + GA	0.56	0.47	83.21	1.08	60.00
CDH + PEI	0.56	0.53	94.29	0.79	44.89
CDH + GEN	0.56	0.55	97.86	1.77	100.00
LAC + GA	0.03	0.00	0.00	0.00	0.00
LAC + PEI	0.03	0.00	14.29	23.42	100.00
LAC + GEN	0.03	0.01	35.71	0.00	0.00

**Table 2:** Analysis of the Amide I band of CDH and laccase, a component of secondary structures, band positions, and % contribution in the Amide I band.

Enzyme	Secondary Structure	Position [cm^−1^]	% of Area
CDH	Antiparallel β-sheet	1697	0.75
	Turns and loops	1682	21.02
	3_10_-helix	1663	23.95
	α-helix	1645	29.78
	β-sheet	1628	23.60
	Aggregated	1608	0.47
laccase	Antiparallel β-sheet	1689	0.68
	Turns and loops	1676	4.83
	α-helix	1657	77.96
	β-sheet	1635	13.35
	Aggregated	1624	2.59

**Table 3:** Influence of the activating substances on the Amide I band of CDH and laccase.

Enzyme	Activating Substance	Structure	Position [cm^−1^]	Rise [+]/Loss [34]
CDH	PEI	Turns and loops/Anti β	1683	-
		Turns and loops	1668	-
		3_10_-helix	1660	-
		α-helix	1653	-
		α-helix	1641	+
		β-sheet	1629	+
		Aggregated	1618	+
	GEN	Antiparallel β-sheet	1693	-
		Turns and loops	1682	-
		3_10_-helix	1668	-
		α-helix	1646	+
		β-sheet	1627	+
		Aggregated	1611	+
	GA	Antiparallel β-sheet	1691	+
		Turns and loops	1681	+
		3_10_-helix	1668	-
		Unordered	1656	-
		α-helix	1647	-
		α-helix	1640	-
		β-sheet	1626	+
		Aggregated	1616	+
laccase	PEI	Antiparallel β-sheet	1689	-
		Turns and loops/Anti β	1681	-
		Turns and loops	1671	-
		α-helix	1652	-
		β-sheet	1641	+
		Aggregated	1625	+
	GEN	Antiparallel β-sheet	1691	-
		Turns and loops	1675	-
		3_10_-helix	1661	-
		α-helix	1646	+
		β-sheet	1633	+
		Aggregated	1616	+
	GA	Turns and loops/Anti β	1683	+
		Turns and loops	1674	+
		3_10_-helix	1665	+
		Unordered	1656	-
		α-helix	1647	-
		β-sheet	1637	-
		β-sheet	1621	+
		Aggregated	1612	+

**Table 4:** Lactose conversion efficiency and qualitative analysis of the resulting product (lactobionic acid) in different enzymatic systems using HPLC chromatography.

Enzymatic System	Lactobionic Acid Concentration [mM]	Conversion Efficiencies [%]
Control PchCDH + CuLAC	22.66	45.32
PchCDH/GA + CuLAC	8.32	14.07
PchCDH/PEI + CuLAC	43.01	93.34
PchCDH/GEN + CuLAC	14.80	26.14
CuLAC/GA + PchCDH	4.35	8.38
CuLAC/PEI + PchCDH	1.71	3.57
CuLAC/GEN + PchCDH	5.21	10.34
PchCDH/GEN + CuLAC/GEN	0.00	0.00
PchCDH/GEN+ CuLAC/PEI	4.76	10.23
PchCDH/GEN + CuLAC/GA	0.24	0.51
PchCDH/GA+ CuLAC/GEN	0.00	0.00
PchCDH/GA + CuLAC/PEI	0.84	1.81
PchCDH/GA+ CuLAC/GA	0.00	0.00
PchCDH/PEI + CuLAC/GEN	0.74	1.41
PchCDH/PEI + CuLAC/PEI	9.81	22.34
PchCDH/PEI + CuLAC/GA	0.00	0.00

## Data Availability

Data sharing not applicable: No new data were created or analysed in this study. Data sharing is not applicable to this article.

## Supplementary Materials

The following are available online at https://www.mdpi.com/article/10.3390/jfb14070383/s1, Figure S1. A qualitative study of lactobionic acid using the TLC method. The samples included free laccase and immobilised cellobiose dehydrogenase (A), free PchCDH and immobilised CuLAC (B), and two immobilised enzymes on media activated with different crosslinking agents (C, D, E). The control samples included 25 mM lactose in acetate buffer (lanes 1) and commercial LBA (cLBA) at a concentration of 20 mM (lanes 2). The test samples were as follows: PchCDH/GA and free CuLAC (lane 3), PchCDH/PEI and free CuLAC (lane 4), PchCDH/GEN and free CuLAC (lane 5), CuLAC/GA and free PchCDH (lane 6), CuLAC/PEI and free PchCDH (lane 7), CuLAC/GEN and free PchCDH (lane 8), PchCDH/GEN and CuLAC/GEN (lane 9), PchCDH/GEN and CuLAC/PEI (lane 10), PchCDH/GEN and CuLAC/GA (lane 11), PchCDH/GA and CuLAC/GEN (lane 12), PchCDH/GA and CuLAC/PEI (lane 13), PchCDH/GA and CuLAC/GA (lane 14), PchCDH/PEI and CuLAC/GEN (lane 15), PchCDH/PEI and CuLAC/PEI (lane 16), and PchCDH/PEI and CuLAC/GA (lane 17). Figure S2. The type of the cluster and the number of particle aggregates in the cluster were obtained with the GA (glutaraldehyde) cross-linker. A—chitosan particles (*control*); B—chitosan activated with 5% GA; C—chitosan activated with 5% GA and with immobilised CDH; D—chitosan activated with 5% GA and with immobilised LAC. Figure S3. The type of the cluster and the number of particle aggregates in the cluster were obtained with the PEI (polyethyleneimine) cross-linker. A—chitosan particles (*control*); B—chitosan activated with 0.5% PEI; C—chitosan activated with 0.5% PEI and with immobilised CDH; D—chitosan activated with 0.5% PEI and with immobilised LAC. Figure S4. The type of the cluster and the number of particle aggregates in the cluster were obtained with the GEN (genipin) cross-linker. A—chitosan particles (*control*); B—chitosan activated with 0.5% GEN; C—chitosan activated with 0.5% GEN and with immobilised CDH; D—chitosan activated with 0.5% GEN and with immobilised LAC.
