# Supplementary material for: Immobilisation of Cellobiose Dehydrogenase and Laccase on Chitosan Particles as a Multi-Enzymatic System for the Synthesis of Lactobionic Acid
Source: J Funct Biomater. 2023 Jul 21;14(7):383. doi: 10.3390/jfb14070383 (PMC10381469; doi:10.3390/jfb14070383)
Supplement: Supplementary file 1 [file jfb-14-00383-s001.zip › jfb-2480478-supplementary.pdf]

Supplementary information

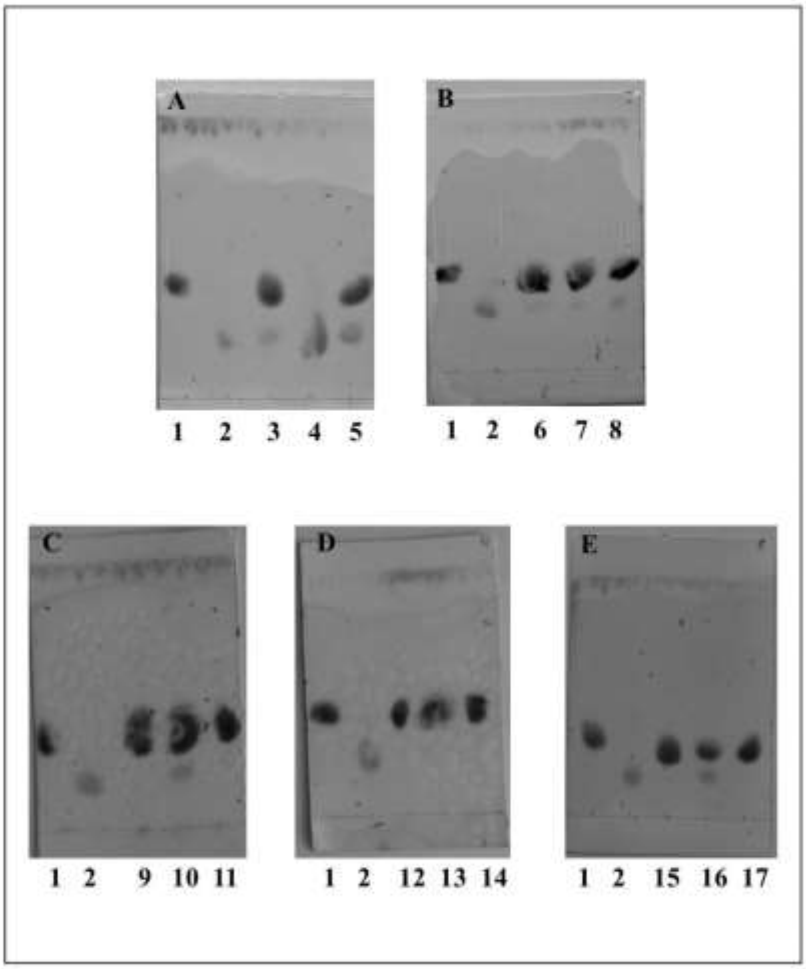

Figure S1. Qualitative study of lactobionic acid using the TLC method. The samples included free laccase and immobilised cellobiose dehydrogenase (A), free PchCDH and immobilised CuLAC (B), and two immobilised enzymes on media activated with different crosslinking agents (C, D, E). The control samples included 25 mM lactose in acetate buffer (lanes 1) and commercial LBA (cLBA) at a concentration of 20 mM (lanes 2). The test samples were as follows: PchCDH/GA and free CuLAC (lane 3), PchCDH/PEI and free CuLAC (lane 4), PchCDH/GEN and free CuLAC (lane 5), CuLAC/GA and free PchCDH (lane 6), CuLAC/PEI and free PchCDH (lane 7), CuLAC/GEN and free PchCDH (lane 8), PchCDH/GEN and CuLAC/GEN (lane 9), PchCDH/GEN and CuLAC/PEI (lane 10), PchCDH/GEN and CuLAC/GA (lane 11), PchCDH/GA and CuLAC/GEN (lane 12), PchCDH/GA and CuLAC/PEI (lane 13), PchCDH/GA and CuLAC/GA (lane 14), PchCDH/PEI and CuLAC/GEN (lane 15), PchCDH/PEI and CuLAC/PEI (lane 16), and PchCDH/PEI and CuLAC/GA (lane 17).

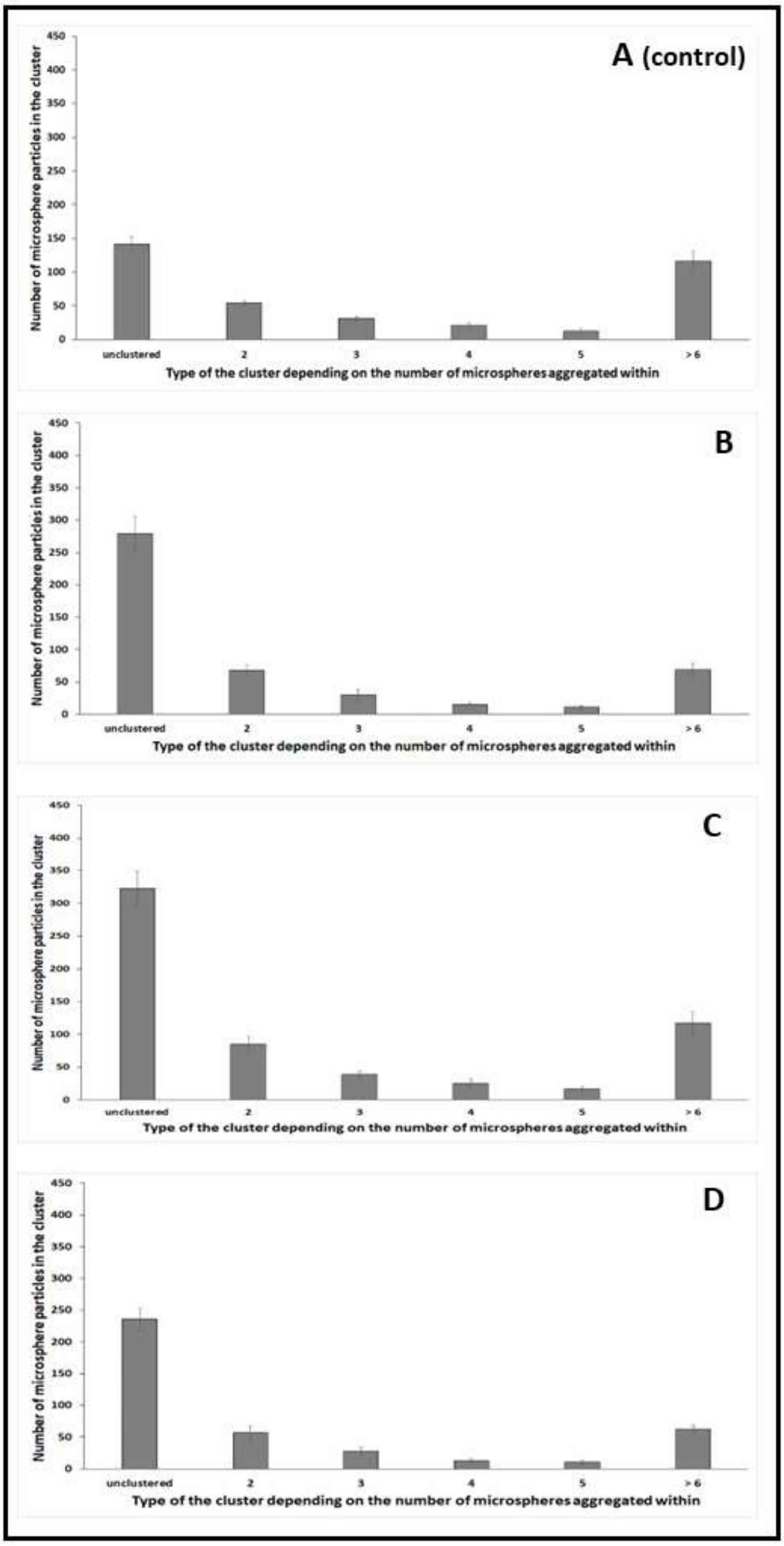

Figure S2. The type of the cluster and the number of particle aggregates in the cluster obtained with the GA (glutaraldehyde) cross-linker. A – chitosan particles (*control*); B – chitosan activated with 5% GA; C - chitosan activated with 5% GA and with immobilised CDH; D - chitosan activated with 5% GA and with immobilised LAC.

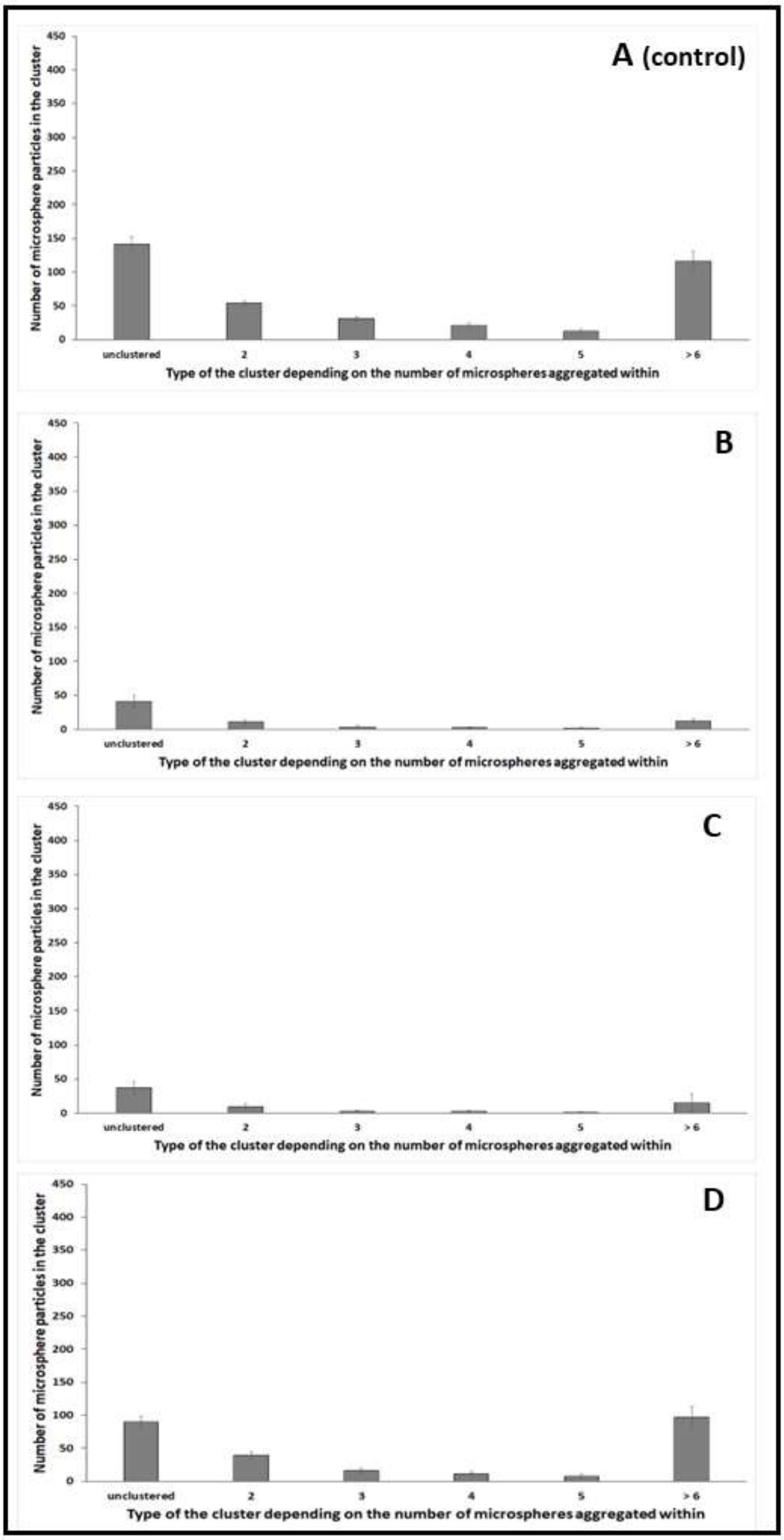

Figure S3. The type of the cluster and the number of particle aggregates in the cluster obtained with with the PEI (polyethyleneimine) cross-linker. A – chitosan particles (*control*); B – chitosan activated with 0.5% PEI; C - chitosan activated with 0.5% PEI and with immobilised CDH; D - chitosan activated with 0.5% PEI and with immobilised LAC.

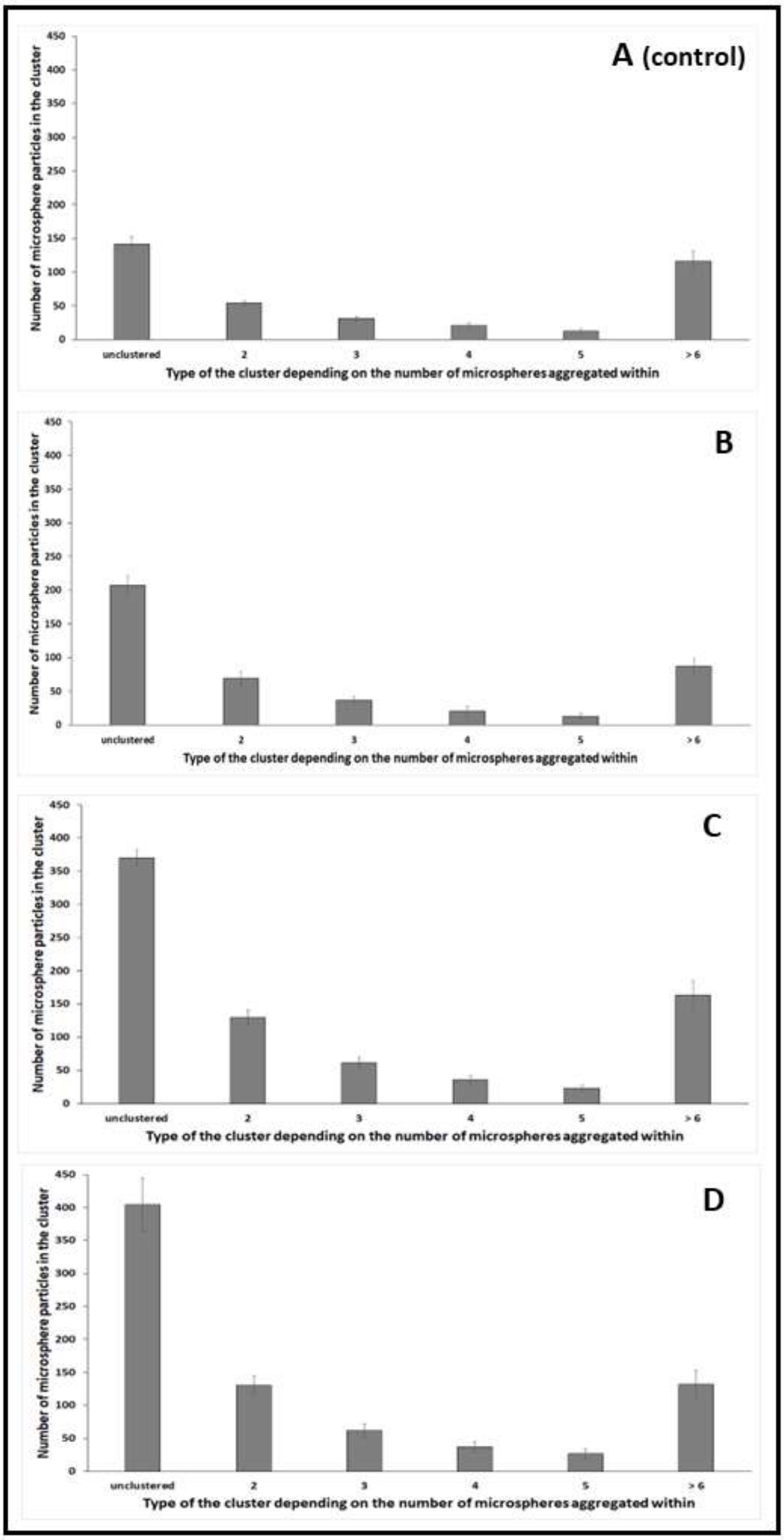

Figure S4. The type of the cluster and the number of particle aggregates in the cluster obtained with with the GEN (genipin) cross-linker. A – chitosan particles (*control*); B – chitosan activated with 0.5% GEN; C - chitosan activated with 0.5% GEN and with immobilised CDH; D - chitosan activated with 0.5% GEN and with immobilised LAC.
